# Dr. Drake’s Interrogatories

**Published:** 1853-01

**Authors:** 


### DENTAL REGISTER OF THE WEST.

VOL. VI. CINCINNATI, JANUARY, 1852. No. 2.

### Original Communications.

### *Dr. Drake's Interrogatories.*

The following letter was received by the Mississippi Valley Association of Dental Surgeons at their annual meeting, Sept. 2d, 1846. The committee, as at first organized, never had an opportunity for consultation and have never reported. At the annual meeting, in Sept. 1850, the committee was re-organized, and their report has been delayed, mainly because Dr. Drake was not in readiness to use the report, and they intended placing it in his hands before its publication. At the last meeting of the society it was suggested that the report be published in the Register, as soon as prepared, since which time we had an interview with Dr. Drake, and this plan being in accordance with his views, we commence, in the present number of the Register, its publication. It is but due to the other members of the committee to remark that the report, thus far published, has not passed through their hands as it was intended. The distance which seperates the committee precludes much consultation. We have requested them to prepare answers to some of the questions and they will appear in their regular order and indeed any further remarks they may see proper to offer on the first question. The committee cannot but deplore the decease of the eminent individual, who, in his zeal for the promotion of all which relates to medical science propounded these questions, their vast comprehension and direct application to the subject which he had in view (the decay of the dental organs) shows the power and grasp of that mind which grappled as a man of strength with his favorite science. Our association of which he was an honorary member, and the medical public mourn his loss. We are glad to see that his unfinished work will be put in proper hands for publication at an early period. The report—which we regret is now too late for his revision—will be placed in the hands of whoever takes charge of his papers, for such use as may seem best.

JAMES TAYLOR.

---

CINCINNATI, SEPTEMBER 2nd, 1846.

*To the President of the Mississippi Valley Association of Dental Surgeons.*

SIR:—In the historical and practical treatise on our diseases, which I am now engaged in preparing for the press, I have appropriated a chapter to the maladies of the teeth; and am anxious to obtain, from gentlemen devoted to their treatment, as much information as possible. Permit me, then, to request of the members of the association, over which you preside, such facts and observations as they may be able to communicate, on the following points:

1st. What is the nature of that diathesis, or constitutional predisposition or disorder (if any,) which so often occasions decay in the deciduous teeth of our children?

2nd. To what causes, external or pathological, local or constitutional, shall we ascribe the premature decay of the second teeth, in the west?

Is a hereditary, scrofulous diathesis a cause of infirm teeth?

Is dyspepsia a cause of early decay?

Does the acid thrown up by many dyspeptics, in paroxysms of that disease, act chemically on the teeth?

What is the effect of repeated salivations, on the teeth and gums?

What are the effects of tobacco on the teeth, and are those of chewing and smoking the same?

3rd. Has the tarter of the teeth a constitutional origin?

4th. Is the decay of teeth greater in the west, than in the Atlantic States, in the same latitudes; and is there any difference, in different latitudes, in the same meridians?

5th. Is there any difference as to soundness of teeth, between our native and foreign population?

6th. Are the teeth of our colored people, less subject to decay than those of white, who labor and live in a similar manner, as to diet and drinks?

Replies to these questions, or information (not referred to in them) on diseases of the mouth generally, communicated to me within the next few months, will be acknowledged as a favor, while I shall scrupulously give with every new or important observation, the name of its author.

I have the honor to be, very respectfully,

Your obedient servant,

DANIEL DRAKE.

---

PROF. DANIEL DRAKE, M. D.—DEAR SIR:—The interrogatories which you put to the Mississippi Valley Association of Dental Surgeons in relation to the maladies of the teeth, were first submitted to a committee of three, viz: Drs. J. W. Cook, W. E. Ide, and James Taylor. The two former gentlemen having withdrawn from the Society; in the fall of 1850, at our annual meeting the committee was re-organized by the appointment of Drs. W. H. Goddard and A. Berry in their place. The committee have delayed their answers to your questions, first, because they understood from yourself that the volume in which you designed inserting the chapter on Maladies of the Teeth, would not go to press before 1853. And second, because we felt that they required much careful investigation and study. Your first two questions embrace the entire foundation of dental science. That which has engaged the attention of our best writers, and on which there has been much diversity of opinion. It is, therefore, with some hesitancy we approach this subject. We are aware that the Dental Profession, of late, as a body, have generally taken different views from those most generally received by the Medical Profession. This will render it necessary that we go into a more full and lengthy investigation of, particularly your two first interrogatories. These two questions are so intimately blended that when the first is answered but little remains to be said on the other. Both relating to the cause of decay.

You first ask, "What is the nature of that diathesis or constitutional predisposition or disorder (if any,) which so often occasions decay in the deciduous teeth of our children?"—This question, but in a much less philosophic manner, is almost daily forced upon us. "Why is it our children's teeth decay so soon?" Our attention is thus daily drawn to the little sufferers. Some are ruddy and strong—some weak, feeble, and sickly. Some, at the early age of three or four years, have lost the crowns of many of their teeth, others show less disease, but few none. Every dental practitioner will fail to answer this question satisfactorily to himself and the parent who asks it, by attributing it solely to the food on which these children respectively live. He may not be able to determine in this any material difference. Neither is the difference traceable to that care which is bestowed by one more than the other. For a large majority of the cases to which our attention is drawn, have received very similar attention as it regards cleanliness. Not but that we regard cleanliness as of the utmost importance—but we wish to draw attention to that constitutional defference which is everywhere so manifest, and to which an answer is sought in your question. Man's constitutional development receives its impress almost as soon as fœtal life is apparent—a peculiar constitution, with its appropriate diathesis, exhibits as early in life as can be traced, certain and marked characteristics, and in no part of our organization is this more apparent than in the organs of mastication.

These physical characteristics may not be, it is true, so well marked in the deciduous teeth as in the permanent organs; yet to some extent they exist, and form one of the marks with the Dentist in determining the constitutional predisposition of the individual.

But in determining the causes which operate to produce an imperfect or enfeebled dental organization, we are led to that period of utero gestation in which we find the first development of these organs. For much of our knowledge on this subject we are indebted to the researches of Arnold, Goodsir, and Nasmyth. In January 1839, Mr. Goodsir gave us in the Edinburgh Medical and Surgical Journal the result of his researches, from which we gather, that as early as the "seventh week after conception the germ of the first temporary molaris in the upper jaw may be seen." These germs or small papillæ which are the rudiments of the deciduous set, have all made their appearance by the end of the tenth week, and as early as the thirteenth week they are enclosed in a follicle, and begin to assume a particular shape indicative of the tooth which is to be formed, and by the fifth month ossification has commenced. By the time of birth the crowns of these teeth are formed, and the roots begin to be formed. In the earliest period of their development, we find them taking their position in a groove formed for their reception. This groove is lined with the mucous membrane, and the processes sent off to enclose the germ of the future tooth in a follicle and sac are sent from the inner and outer lips of this groove, and maintain their mucous covering, so that the sac itself is lined with the same membrane.

Blandin, in his Anatomy of the Dental System remarks that "the teeth are a production of the internal tegumentary system; they are true appendages of the digestive membrane, in a depression of which they are lodged by their adherent extremities."

At birth, then, we have these teeth so far as their crowns are concerned, pretty well developed, and if defective in their organization we must look to the constitutional diathesis of the parents, and especially the mother, and so far as causes may operate on her to those which may effect the mucus secretions, and the quality of the blood which supply the materials for the production of these organs, and hence, as hidden and mysterious as the operations of the animal economy are, yet enough has been elucidated to serve as guides in the investigation of this subject. It has been remarked by one of our best writers on Animal Chemistry that "the transition from healthy to diseased mucus is so indefinately characterized, that it is almost impossible to draw a strict line of demarkation between them." The secretion is however, in a normal state, alkaline, and maintains this generally even in a morbid condition. In the latter state, however, the epithelium cells are diminished, and the constituents of Soda, Potash and Silica are gone.* This, however, is from the analysis of Nassa and Gruby of the mucus of the bronchical and pulmonary secretions; like changes, however, occur in all the mucus secretions which have been analysed, and we allude to it now to show the effect which may result from the ever varying changes which take place in the secretions of this membrane on the organs which germinate and are matured from this source.

It is a generally received, and we believe an established fact, that the health and well-being of the fœtus in utero, is effected by the condition of the mother. Hippocrates observ-

---

* It may be thought that these changes could not exert an influence on the development of the teeth. But we would throw out the following suggestions: The secretions of which we are speaking are from the mucus membrane.—The germ of the teeth are deposited on this membrane, and the first indication we have of the rudiments of the dental organs are small granular deposits or elevations on this membrane. Simon remarks, that examined under the microscope mucus "is found to consist of a liquid in which minute rounded or prolonged corpuscles of a granulated appearance (mucus corpuscles,) are enclosed." And he further remarks that "the transition of mucus corpuscles into epithelium cells may be easily seen." And again, that this fluid is pervaded by a "finely granulated substance." To what extent this granular substance resembles that which is the primitive dental organ, we can only conjecture; but this we do know, this seeretion contains in a healthy state some of the same constituents which are found in the teeth.

ed that "the health of the child was conformable to that of the mother." And there can be no doubt that the germs of these teeth are often seriously injured during their development. These teeth are all matured from that nourishment supplied by the mother—either before or after birth. Independent of a physiognomic trait or a constitutional tendency which a child may receive from its father—its nourishment and support must be derived from its mother. As she is nourished and sustained, so is the child, and if that nourishment which would impart proper material for the perfect development of the teeth is withheld, it is impossible that they should receive perfect organization. Those organs, unlike the osseous system in general, and the muscular, are not reproduced, or an injury inflicted in their production, cannot be repaired, at least to any great extent. This is particularly true as it regards the enamel; as the child advances in life the dentine becomes more dense and perfect, and better able to resist those agents which are calculated to destroy them. This is owing to an increased deposition of the earthy particles.

The varied hereditary tendencies to particular forms of disease which are everywhere manifest to careful observation, also show that those children born during the time the parent or parents have been most under the diseased influence, possess a more enfeebled constitution than those born when in better health. We believe that certain forms of disease which scarcely manifest itself during early life, when both parents are in the prime and vigor of life, give also a better constitution, and with it dental organism to their children, than in a later period of life. We have seen this so often illustrated in numerous families that we cannot but regard it as a strong argument that good or defective organization of the teeth depend very much on the constitution of the parents, and the condition of the health of the mother during gestation. There are certain elements entering into the constituents of the solids and fluids of the body which must be gathered from the aliment taken by the mother during pregnancy, and also the period of lactation, and after this from that nourishment which the child receives from other sources. This being the fact, it is obvious, then, that as the secretary system is supplied with materials out of which to form the several organs which make up he general organization, so will this be accomp'ished; as before remarked, the teeth are products from the mucus membrane, and this is so impressable that it has been considered difficult to determine what is really the normal condition of its secretions. It is not, however, difficult to determine the ultimate product of the secretion which develope the teeth—we have here a large preponderance of the phosphate of lime.

The blood appears to be the great element out of which the animal body is fabricated, and it is evident that diet and disease exert on this fluid very important changes. Lecanu, who has made repeated analyses of the blood under different conditions of health, temperament, &c., remarks "that temperament has an influence upon the composition of the blood, and that the blood of lymphatic persons is poorer in solid constituents, and especially in blood corpuscles, than that of persons of sanguenous temperament." Age exerts also a difference—the blood of young persons containing a larger proportion of the solid constituents and blood corpuscles, and as might be expected, the blood of the fœtus is found much richer in these constituents than the mother; and as an illustration of this, we present the result of two analyses of Denis—one of the "venous blood of the mother, the other of the placental blood as it issued from the artery of the cord."

| | *Venous blood of the Mother.* | *Blood of Umbilical Artery.* |
|---|---|---|
| Water, - - | 781.0 | 701.5 |
| Solid residue, | 219.0 | 298.5 |
| Fibrin, - - | 2.4 | 2.2 |
| Albumen, - | 50.0 | 50.0 |
| Blood Corpuscles, | 139.9 | 222.0 |
| Per. Oxide of Iron, | 6.8 | 2.0 |
| Phosphorised fat, | 9.2 | 7.5 |
| Osmazone & Cruozin | 4.2 | 2.7 |
| Salts, - - | 12.5 | 12.1 |

Here we find a marked difference in the solid constituents, and also blood corpuscles, showing an evident arrangement to meet the demands of the fœtus in that development which is going on in the production of the various organs of the system, and particularly the osseous. It is remarked by Simon "that the mass of the blood in the fœtus increases in a very rapid ratio with the development. The proportion of corpuscles is more augmented, and the quantity of water less than occurs at any subsequent period of life." This appears to continue for some time after birth. The experiments of Denis and others confirm these observations.

The quantity and quality of the blood, therefore, must, we suppose, exert a vast influence in the organization of the teeth, the constituents of which are derived from this source. From the view we have here taken, we cannot but regard the fact apparent that there is an inherent diathesis tending to an enfeebled or imperfect dental organization. What, then, you ask is, that "predisposition or disorder which so often occasions decay in the deciduous teeth of our children?" We would reply, that predisposition or diathesis which most poorly supplies the elements for their production. These elements are more efficiently supplied in a state of health by that general constitution which is denominated sanguinous or sanguino-bilious, and most poorly supplied by that termed lymphatic or lymphatico-serous. In the latter we have less of the elements of the blood out of which the calcarious ingredients of the teeth are fabricated, and it is now a well established fact that bones acquire solidity no faster than the parts which are about to ossify, become charged with red blood."* It has been remarked, also, by Delebarre and others, that "the superficial layer of the pulp reddens before it ossifies.

The result of Gruelin's analyses of the lymphatic fluid, the predominence of which determines the constitutional diathesis, is a little remarkable as bearing on this point.

His table is as follows: In 1000 parts of human lymph be found—

*The researches of Duhamel and Haller confirm this—see Harris' Principles and Practice of Dental Surgery—page 199.

| | |
|---|---|
| Water, - - - - - - - | 961.0 |
| Solid Constituents, - - - | 39.0 |
| Fibrin, - - - - - - - | 2.5 |
| Albumen, - - - - - - | 27.5 |
| Chloride of Sodium Phosphates of Potash and Soda, and salivary matter, - | 2.1 |
| Extractive matters and lactate of soda, | 6.9 |

Let us make two or three comparisons here—taking the blood of the umbilical artery, as in our former table, and compare the water and solid constituents.

In the blood of the umbilical artery we have in 1000 parts,

| | | | |
|---|---|---|---|
| Water, | 701.5, | In the Lymph, Water, | 961.0 |
| Solid residue, | 298.5, | Solid residue, | 39.0 |

and in the salts as great a difference relatively. It is very easy to deduce from this presentation of the subject the effect which would naturally be produced on the teeth in their formative state, by that condition of system which we would call of the most perfect order of sanguine temperament, and also that which would result from the one strictly lymphatic. This, however, relates merely to the most healthy development of either of these constitutions. The blending of the sanguinous with the lymphatic, and with the other temperaments, would, as a matter of course, modify very much the condition of these organs, as well as all the other organs of the system, deteriorating more or less their innate constitutional vigor; so, likewise, the blendings of the lymphatic with the sanguinous and bilious, nervous, &c., would materially improve it, and particularly the more it received from the sanguinous. Thus much in relation to that part of your question determining if there is a "constitutional predisposition or disorder, (if any,") tending to the early decay of the deciduous teeth.

Our answer is in the affirmative, and that the constitutional predisposition would be most appropriately classed the lymphatic temperament, or what Lafourge calls lymphatico-serous.

In answer to your first question we might stop here—but in closing your interrogatories you remark "that replies to these questions, or information (not referred to in them,) on diseases of the mouth generally, will be acknowledged as a favor," &c. In connection with this question, however, we would further draw attention to other conditions which may be the result of disease. The Dental Practitioner needs as much a correct knowledge of Pathology as Physiology, so that when he sees a healthy operation of the economy has been disturbed, he may trace out the cause and apply, so far as possible, the remedy.

We have taken the most favorable, and also the most unfavorable constitutional diathesis. Showing the most perfect, and perhaps the most imperfect constitutional development as it regards the teeth—having the system, however, in its most healthy operation under both developments. In one case we should expect a very perfect denture, and in the other an enfeebled dental organization. Yet even here, uninfluenced by diseased action, the teeth might have a tolerable constitution, and under favorable circumstances be preserved for life. Such teeth, however, would never resist so effectually those agents which are calculated to decompose them; hence, those diseases affecting the secretions of the mouth would most likely exert an injurious influence, while the same degree of vitiation would not affect the former.

If under the most healthy operations of an enfeebled constitutional development, we have an imperfect dental organization, what might we expect when this development takes place under diseased action in the same constitution? If certain diseases affect the vigor of fœtal life, and bring into the world children scarce able to endure the first impressions of a new existence, with every organ so enfeebled that life, for a time, is a continued struggle, in what condition might we expect those organs elaborated from the delicate mucus membrane?

In connection with this subject, it will be interesting to trace out some of the pathological conditions of the blood during disease. Deviations from its normal condition have long been noticed by pathologists. Dr. Simon remarks that "the quantity of the fibrin is sometimes found to be very much increased, while in other cases it is present only in such very small proportions that no clot is formed. The blood is sometimes found to be very "rich in solid constituents, and especially in blood corpuscles; while at other times it will be so poor as to resemble colored water."

These deviations from the healthy condition whether affording more or less of the elements which enter into the composition of the teeth, may be still prejudicial to their perfect development, for a diseased secretory organ could scarce perform a healthy function; yet when there is evidently a great want ot the proper elements (and sometimes scarce a trace left,) this function must be defective.

In "phlogoses of the circulating system," as a general rule, hyperinosis exists. The fibrinous portion of the blood increasing, and as this takes place the blood corpuscles decreasing in amount. This hyperinosis is manifest in all inflammatory diseases, and especially in pneumonia, at times the blood corpuscles appear to be diminished near one-half, and the fibrin proportionally increased. Rheumatism (acute,) exhibits about the same results. In phthisis tuberculosa the fibrin is increased, and the blood corpuscles decrease and this in proportion to the progress of the disease.

In hypinosis of the circulating system we have a diminished amount of fibrin, and the maximum of the blood corpuscles is either maintained or increased; this is observable in typhus abdominalis, febris continua, varioloid, &c. "In scrofulous affections the blood is deficient in solid constituents." We shall not follow out, however, the different pathological conditions of the blood in different forms of disease. The changes in the constituents of this fluid are numerous, depending on the amount of increased inflammatory action, progress of disease, loss of blood by repeated venesection, &c., &c.

Those diseases, however, which subtract the largest amount of the elements for the production of the osseous structure, might naturally be supposed to affect the most injuriously the organization of the teeth, hence, inflammatory diseases would be placed in this order. On a closer inspection, however, this would not necessarily be the case.

"It has been suggested that blood in which there is an excess of fibrin increases the energy of the hearts action, while blood deficient in fibrin diminishes it. The rapid circulation of the blood in inflammations, and its torpid condition in certain typhoid affections seems in favor of this view." Dr. Simon also remarks that "observations have shown us that blood retained for any length of time in an organ, and thus prevented from meeting with a due supply of oxygen, becomes poorer instead of richer in fibrin, whereas there is undoubted evidence that in inflammation the fibrin is increased. Moreover, blood impeded in the course of the circulation becomes darker, (a sign that there is not a due supply of oxygen,) while blood in inflammation is generally brighter than in the normal state. He further remarks that "we can, I think, scarcely doubt that the blood in an inflamed organ differs in its composition from the blood in the rest of the body, provided that we can assume that there is a stagnation of the blood in the affected organ during the whole period of inflammatory action." It is a well established fact that as ossification is about to commence we have an increased supply of blood to the part—the layer of bone about to be formed is first reddened by this, and as the calcarious ingredients are deposited this is removed, leaving the bone white. If, then, during this operation of the economy we have increased arterial action as the result of general inflammation, we have also thrown to this part a greater amount of blood, and although poorer in the elements for the elaboration of these organs, yet this poverty may be made up for by the increased supply and the increased action of the secreting organs, this increased supply of blood and rapid elaboration of the elements for the tooth's production might prevent much injury so long as the inflammation was not seated directly in the organ itself, or did not materially change the alkaline condition of the secretions of the mucus membrane.

If, however, inflammation should be seated in the organ for the tooths development, and "stagnation of blood" be the consequence, we would have obstructed deposition of earthy ingredients, or these imperfectly deposited.

In hypinosis of the blood where we have a deficiency of fibrin, and often an increased supply of blood corpuscles, we may have as in typhus abdominales, fibrin continua, varioloid, &c., a torpid condition of the circulation (and this is perhaps more apparent in the capillary circulation,) we would have then a loss of vital action, and an obstructed elimination of the elements for the tooths production. When it is remembered that the tooths germ is attached to the mucus membrane and receives its nourishment through this delicate and impressable membrane, it is not more than rational to conclude that the long continued derangement of the secretions, as in many of our protracted forms of disease, would seriously obstruct the perfect development of these organs.

In the production, however, of the deciduous organs, there is only a short period when this operation of the economy of the system is subject to such influences, except through the mother.* There can be no doubt but that disease effecting the mother, must exert an influence on the child in utero, and that the dental organization may be as much injured thereby as any part of the system.

The subject of nutrition is so intimately connected with this investigation that we feel we shall be excused for briefly alluding to it. And first let us notice the effect on the mother, and thus to the child, and then after birth on the child itself. We have shown that the blood is very much changed in its character by different diseases; may we not expect changes also in this fluid by nutritious and unnutritious diet. There must be eliminated from the aliment taken into the system for nourishment two essential principles; one to nourish, and the other to heat the body. Those articles of food which best meet this demand of the animal economy will best secure a perfect organization. By the act of digestion three constitu-

* The crowns of the deciduous teeth are formed at birth, yet the last molar of this set sometimes does not appear through the gum until the 20th month.

ents are prepared for the production and reparation of the system.

These are albumen, saccharine and oleaginous matter. In the albumen we have the nutrient principle and the calorifacient in the saccharine and oleaginous.

Both these principles are essential to animal life, yet we may have too much of the one and not enough of the other, and those articles of food which supplies but poorly the nutrient principle would fail in giving due nourishment to the system.

From an essay, from James Paul, M. D., on the food and the teeth, read before the "District Medical Society of the county of Mercer, and published in the N. J. Medical Reporter, Dental Register, &c. We take the following table, showing the elementary constituents of these two essential principles, as found in eggs, wheat, sugar, mutton, &c., &c.:

| | *Albumen.* | | *Saccharine.* | | | | *Oleaginous.* |
|---|---|---|---|---|---|---|---|
| | Eggs. | Wheat. | Starch Arrow root. | Sugar from Starch. | Sugar of Milk. | Cane Sugar. | Fat. |
| Carbon, | 55·000 | 55·01 | 44·40 | 37·29 | 40 00 | 42·301 | 78·996 |
| Hydrogen, | 7·073 | 7·23 | 6·18 | 6·84 | 6·61 | 6·384 | 11·700 |
| Nitrogen, | 15·920 | 15 92 | | | | | |
| Oxygen, Sulphur, Phosphorus, | 22·007 | 21.84 | 49·42 | 55·87 | 52·93 | 51·315 | 9·304 |

If it be true as Dr. Paul remarks that Nitrogen is "required in those compounds which give strength and formation to the frame." we would find a poor supply from the saccharine and oleaginous compounds.

We make up the following table of the relative nutritive properties of various articles of food, from the same essay:

| | | | | | | |
|---|---|---|---|---|---|---|
| 100 lbs. | wheat bread | contains | 30 | lbs. of | nutritive | elements. |
| " | Flesh | " | 21 | " | " | " |
| " | French Beans | " | 80 | " | " | " |
| " | Peas | " | 83 | " | " | " |
| " | Lentils | " | 94 | " | " | " |
| " | Potatoes | " | 25 | " | " | " |
| " | Carrots | " | 14 | " | " | " |
| " | Beets | " | 8 | " | " | " |

This table shows a great difference in the nutritive properties of those different articles of food, but it would not do to suppose that as peas and lentils contain a greater amount of the nutritive element they would, under all circumstances, be better diet for the mother than wheat bread, for the latter contains a much larger proportion of the inorganic elements (lime &c.) It is instructive here and proper to notice the difference between the constituents of wheat before the bran is seperated and the flour itself.

Prof. Johnston's analyses is as follows:

| | | *Muscular Matter.* | *Fat Prin.* | *Bone & Sal.* |
|---|---|---|---|---|
| In 1000 lbs of whole grain, were | | 156 lbs | 25 lbs | 170 lbs |
| " Fine Flour, | " | 120 " | 20 " | 60 " |
| " Bran, | " | | 60 " | 700 " |

These three substances taken together, according to the same authority, show the following result:

| | *Whole Grain.* | *Fine Flour.* |
|---|---|---|
| Of muscular matter, | 156 lbs | 130 lbs |
| Of bone material, | 170 " | 60 " |
| Of fat, | 25 " | 20 " |
| | 351 | 210 |

Showing that the whole grain is far more "nutricious than fine flour," and also more than double as rich in bone material. The very large amount of inorganic material contained in bran, being 700 out of a thousand parts, exhibit the great advantage, which might be derived from the proper use of this as an article of food. Mothers of a lymphatic temperament or far removed from the sanguinous would certainly consult the good of their offspring, by a proper conformity to such diet during gestation as these analyses would point out. There are many families in whom frail and bad teeth have become an hereditary affliction, whose condition, in this respect as well as in general constitutional vigor we have no doubt might be very mnch improved by a rightly adopted and long continued diet. If this was kept up from generation to generation seeking such localities, also, as would best tend to develope constitutional vigor, we doubt not, we should have an improvement instead of impairment in general health and longevity.

In the essay by Dr. Paul, to which we have already alluded, much valuable information on this subject is given, but it would rather be out of place and it is not our design to enter into a full exposition of this part of our subject, which indeed is only incidental to our reply to your question. But, having alluded to those articles of diet which may give nourishment to the mother and at the same time, afford through her to the child, proper material for a well developed osseous structure during gestation. Let us look at that nourishment, provided by an all wise providence, for the sustenance of the child, until the dental organs are matured and ready for their appropriate use. We allude to the milk, the natural nourishment for the child after birth up to the eruption of the teeth, when different diet is demanded. The Creator here shows infinite wisdom, and as the system is developed makes provision for every exigency.

If we examine healthy milk, we find all the elements requisite to the development of a good organization. The inorganic elements, for the osseous system, that which is necessary for the muscular development and the calorificient, are all here.

Simon gives us the result of fourteen analyses of healthy milk, of different women and of different ages. (We give the maximum and minimum of these analyses.)

| | *Maximum.* | *Minimum.* |
|---|---|---|
| Water, - - - - - | 914·0 | 861·4 |
| Solid constituents, - - | 138·6 | 86.0 |
| Butter, - - - - | 54·0 | 8·0 |
| Casein, - - - - | 45·2 | 19·6 |
| Sugar of milk and centrative matters, | 62·4 | 39·2 |
| Fixed Salts, - - - - - | 2·7 | 1·6 |

The Maximum gives the highest and the minimum the lowest of each constituent. Dr. Paul remarks that "the cheese or casein of the milk" contains the nitrogenised or nutrient principle, which, together with the earth and salts contained in the milk, goes to form the bones, muscles and different tissues of the system. This we see, even in healthy milk sometimes full as low as 19·6. The sugar appears to afford the carbonised principle to sustain the temperature of the child.

Let us now notice some of the changes which this important fluid undergoes. First that of temperament. L'Horetier selected two females of equal age and put them on the same diet and mode of life, one was a fair woman and the other a dark brunette. We give below the result of his analyses:

| | *A Blonde, aged* 22. | | *A Brunette, aged* 22. | |
|---|---|---|---|---|
| Water, - - | 892·0 | 881·5 | 853.3 | 853.0 |
| Solid constituents, | 108·0 | 118·5 | 146·7 | 147·0 |
| Butter, - - | 35·5 | 40·5 | 54·8 | 56·3 |
| Casein, - - | 10·0 | 9·5 | 16·2 | 17·0 |
| Sugar of Milk, - | 58·5 | 64·0 | 71·2 | 70·0 |
| Salts, - - | 4·0 | 4·5 | 4·5 | 4·5 |

Here we notice a marked difference in these 4 analyses, and in each the brunette's milk furnished less water and a much larger amount of solid constituents—butter, casein and sugar of milk; and the inference would be a much better supply of those elements out of which the osseous and muscular system could be fabricated. Let us next see the effect of nutrition, and here Simon had an opportunity of testing this on the same individual, and he remarks, that "it so happened that she was suddenly deprived of the means of obtaining even the most ordinary necessaries of life. The milk secreted at this period (the 11th November,) was sufficiently abundant in quantity, but was very poor in solid constituents, containing only 8·6 0-0. Some days afterwards (the 18th November) she was placed upon a full and nutritious diet. The milk in consequence, was secreted so copiously, as to run spontaneously from the breast; it left 11·9 0-0 of solid constituents." Circumstances again threw her in destitution, when her milk gave again only 9·8 0-0 solid constituents. Again she was put on nutritious diet and her milk gave 12·6 0-0 of solid constituents. We would ask how could it be otherways than that the child nourished, on the milk thus poorly supplied with the nutritive elements, would ultimately show a defect in the perfection of those organs, which, after well feeding, cannot, to any extent, replenish? From the repeated analyses of Simon, during the first six months of childhood, or after delivery we find that the constituent of casein increased, showing an all wise provision for the increasing demand of the system for this element of nutrition and production, while at the same the sugar or the calorificient principle is diminished, the salts are also increased from 0·84 to 2·70.

*Passion.*—It is a well established fact that violent passion exerts a bad influence on this secretion, so much so that children have been suddenly taken with convulsions, diarrhœa, vomiting, &c., by taking the breast while the mother was laboring under the effect of such excitement. The milk analysed at this time has been found to exhibit a marked acid reaction, in its normal state it is alkaline.

*Disease.*—"Meggenhofen found that the milk of a sypilitic woman reddened tincture of litmus," and hence in this state it was of an acid character. Herberger, in his analyses, found that diseased milk furnished the following: water, 895; solid constituents, 105; casein, 18.3; sugar, 28·9; butter, 23·3; chlorides of potassium and soda, lactate and phosphate of potash, and an inorganic substance, insoluble in oil of turpentine, 41·6; organic matter soluble in oil of turpentine 1·6. This, we find, falls below the minimum of healthy milk in casein, and far below the maximum, as given by Simon, in casein, solid constituents and sugar of milk. Deyeux "examined the milk of a woman who was liable to frequent nervous attacks, and found that simultaneously, with these seizures, the milk became transparent and viscid, like albumen, and did not resume its normal condition for some time."

In connection with this subject we think it necessary only to refer you to those particular diseases, which affect children during the period of lactation, some of which are directly seated on the mucous membrane and must necessarily disturb the process of assimilation and prevent the perfect organization of the teeth during this period.

In those constitutional temperaments presenting the most healthy and active mucous tissues, the functional operations of which are not easily disturbed (and such we believe are more frequently met with in sanguineous or bilious temperaments) we have less of those disturbing causes which would impair a perfect development.

In a weak and enfeebled mucous tissue, where the mucous membrane is easily disturbed and when once deranged in its proper function, is slow in regaining its tone and healthy operation, we have that condition most favorable to an imperfect development, and such we think, is most frequently met with in lymphatic temperaments or where we have a lymatico-serous or mucous dyspositions of body, we might also include in this a scrofulous diathesis and particularly where this is blended with a lymphatico-serous temperament.

In lymphatic temperaments, with a scrofulous diathesis, where derangement of the mucous membrane so often terminates in phthisis pulmonalis, frail teeth are a general accompaniment. But this subject will be more directly illustrated when we come to reply to your second interrogatory.

Disease we see may even in the best constitutions impair the perfect development of the dental organization, and these analyses we have of the blood, the milk and various articles of nutrition, point out the necessity for a proper supply of aliment and a perfect and healthy operation of the various functions engaged in the elaboration of the general organization. We cannot see how the process of assimilation can be completed in all its parts, from materials out of which the elements required cannot be procured.

We see that the perfect development of these organs cannot well take place during the operation of disease, which may effect the character of the blood and the condition of the mucous membrane and the secretions therefrom. It may now be asked can disease act thus injuriously after their production? Can organs thus characterized by density of structure, be acted upon by agents elaborated within the buccal cavity, or would not the secretions which are continually thrown upon these organs protect them from the action of any agent which could decompose their structure? Before replying to your second question, which however is intimately associated with this investigation we will draw attention to a few facts bearing on this presentation of the subject.

[TO BE CONTINUED.]

